# River connectivity and climate behind the long‐term evolution of tropical American floodplain lakes

**DOI:** 10.1002/ece3.7674

**Published:** 2021-09-14

**Authors:** Laura Lopera‐Congote, Jorge Salgado, María Isabel Vélez, Andrés Link, Catalina González‐Arango

**Affiliations:** ^1^ Laboratorio de Palinología y Paleoecología Tropical Universidad de los Andes Bogotá Colombia; ^2^ Facultad de Ingeniería Universidad Católica de Colombia Bogotá Colombia; ^3^ School of Geography Nottingham University Nottingham UK; ^4^ Department of Geology University of Regina Regina SK Canada; ^5^ Laboratorio de Ecología de Bosques Tropicales y Primatología Departamento de Ciencias Biológicas Universidad de Los Andes Bogotá Colombia

**Keywords:** climate change, diatoms, floodplains, hydrological connectivity, paleolimnology, tropical rivers

## Abstract

This study presents the long‐term evolution of two floodplains lakes (San Juana and Barbacoas) of the Magdalena River in Colombia with varying degree of connectivity to the River and with different responses to climate events (i.e., extreme floods and droughts). Historical limnological changes were identified through a multiproxy‐based reconstruction including diatoms, sedimentation, and sediment geochemistry, while historical climatic changes were derived from the application of the Standardised Precipitation‐Evapotranspiration Index. The main gradients in climatic and limnological change were assessed via multivariate analysis and generalized additive models. The reconstruction of the more isolated San Juana Lake spanned the last c. 500 years. Between c. 1,620 and 1,750 CE, riverine‐flooded conditions prevailed as indicated by high detrital input, reductive conditions, and dominance of planktonic diatoms. Since the early 1800s, the riverine meander became disconnected, conveying into a marsh‐like environment rich in aerophil diatoms and organic matter. The current lake was then formed around the mid‐1960s with a diverse lake diatom flora including benthic and planktonic diatoms, and more oxygenated waters under a gradual increase in sedimentation and nutrients. The reconstruction for Barbacoas Lake, a waterbody directly connected to the Magdalena River, spanned the last 60 years and showed alternating riverine–wetland–lake conditions in response to varying ENSO conditions. Wet periods were dominated by planktonic and benthic diatoms, while aerophil diatom species prevailed during dry periods; during the two intense ENSO periods of 1987 and 1992, the lake almost desiccated and sedimentation rates spiked. A gradual increase in sedimentation rates post‐2000 suggests that other factors rather than climate are also influencing sediment deposition in the lake. We propose that hydrological connectivity to the Magdalena River is a main factor controlling lake long‐term responses to human pressures, where highly connected lakes respond more acutely to ENSO events while isolated lakes are more sensitive to local land‐use changes.

## Graphical abstract

We present a multi‐proxy approach on short sedimentary cores form two neotropical flood plain lakes associated with the Magdalena River, Colombia, to study temporal variation in diatoms, sediment geochemistry and climate. We proposed that lake hydrological connectivity to the Magdalena River is a main factor controlling lake long‐term responses to human pressures. Highly connected lakes may respond more acutely to prolonged periods of low and high rain, while isolated lakes might be more sensitive to local land‐use changes.

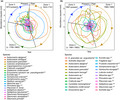

## 1 INTRODUCTION

Tropical floodplain lakes are subject to natural hydrological dynamics imposed by the main river channel and thus are exposed to extreme floods and droughts (Death, 2010; Poff & Ward, 1989; Resh et al., 1988). These hydrological events are known to influence primary productivity and community assembly (Junk et al., 1989), and increase or interrupt ecological connectivity (Amoros & Bornette, 2002), which in turn impacts habitat quality (Lake, 2000). However, natural hydrological dynamics of tropical floodplains can be affected by long‐term (decades–centuries) human‐derived modifications such as river damming, deforestation, land‐use change, and climate change (Angarita et al., 2018; Salgado et al., 2020; Van Looy et al., 2019).

**FIGURE 1:**
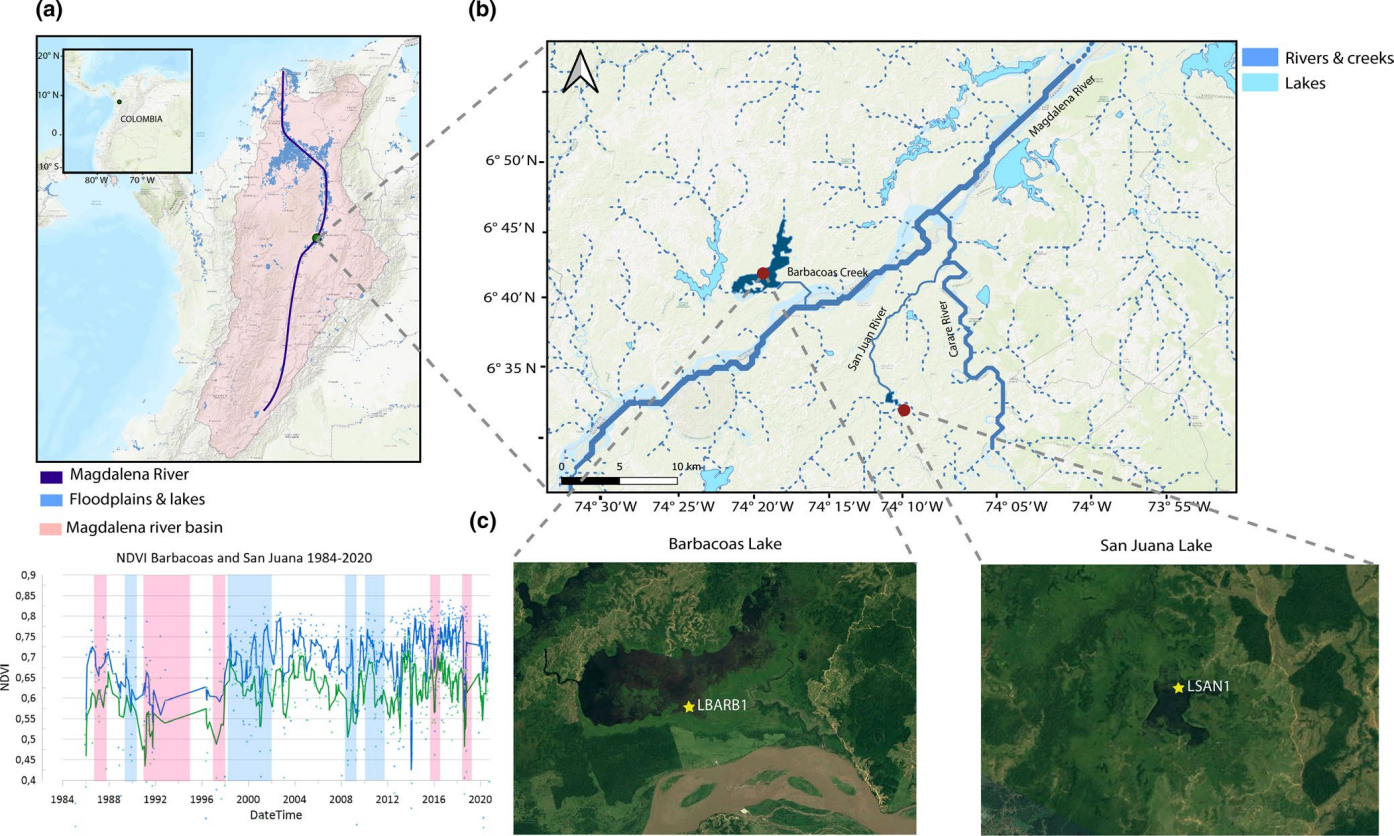
(a) Map of the Magdalena River catchment showing the associated floodplains and lakes (blue) and the location of the study area (green circle); (b) study area showing Barbacoas and San Juana Lakes (dark blue). Connecting rivers are indicated by straight blue lines; (c) aerial zoom into the study lakes showing the coring location for San Juana Lake (LSAN1) and Barbacoas Lake (LBARB1). An NDVI pixel plot is also presented to show the differences in vegetation cover associated with the two study lakes and how it changes according to ENSO. Photographs taken from Goggle Earth (Google Earth V9.132.0.6—WebAssembly with threads. March 19, 2021)

The Magdalena River in Colombia is one of the largest rivers (1,540 km) of South America discharging over 7,100 m^3^/s into the Caribbean Sea and hosting over 70% of the nation’s population and gross domestic product—GDP (Mojica et al., 2006). It dissects the country from south to north, running through the Central and Eastern Andean Cordilleras, producing around 320,000 Ha of floodplains (Figure 1). These offer essential ecosystem services including flood regulation, support, and provision to the local human communities (Montoya & Aguirre, 2009). The river contains one of the largest fish provisions in the region, with key economic species such as the Magdalena catfish (*Pseudoplatystoma magdaleniatum*) and the Magdalena prochilodontid (*Prochilodus magdalenae*; Caballero et al., 2001). Its floodplains, lakes, wetlands, and primary riparian forests are within the Tumbes‐Choco‐Magdalena biodiversity hotspot (Myers et al., 2000) and host endemic birds such as the critically endangered, Blue‐billed Curassow (*Crax alberti*), and other migratory bird species such as the Fishing Eagle (*Pandion haliaetus*), the Yellow‐billed Cuckoo (*Coccyzus americanus*), and the Eastern Kingbird (*Tyrannus tyrannus*; Angel‐Escobar et al., 2014). Other endangered and charismatic vertebrates found in the region include the Brown Spider Monkey (*Ateles hybridus*), the American Manatee (*Trichechus manatus*), the Lowland Tapir (*Tapirus terrestris*), and the River Otter (*Lontra longicauda*; Angel‐Escobar et al., 2014).

Deforestation in the Magdalena River basin has been steadily increasing over the last six decades, with current rates being threefold higher than those from the 1950s (Ayram et al., 2020; Etter et al., 2006; Restrepo & Escobar, 2018). This profound transformation of the landscape has come with a great environmental burden as the river and associated lakes have experienced excess in sediment yields, water pollution, habitat fragmentation, and freshwater fish population declines (Best, 2019; Restrepo, 2015; Restrepo & Escobar, 2018). In addition, more than 20 large dam projects (>20 MW hydropower capacity) across the Magdalena River and tributaries have been constructed or are on their way of implementation resulting in higher fish extinction risks and severe river flow reduction (Angarita et al., 2018; Carvajal‐Quintero et al., 2017).

In large riverine ecosystems, the way in which aquatic communities are organized and respond to climatic or human‐derived stressors largely depends on the degree and magnitude of the disturbance, and on the spatial arrangement of lakes within the main hydrological network (Eros et al., 2012; González‐Trujillo et al., 2020; Grant et al., 2007;). Connected lakes to the main river channel may be for instance, more dependent on the hydrological dynamics of the main river, and hence be prone to greater biological resilience and recovery through source–sink dynamics than in isolated lakes (Salgado et al., 2019). In turn, isolated lakes are likely to present lower dispersal, and thus, local environmental change such as nutrient inputs (eutrophication) is likely to exert a greater control over the community structure through temporal species turnover (Salgado et al., 2019). However, increased habitat connectivity may also disrupt ecosystem resilience by homogenizing lake communities (Strecker & Brittain, 2017). A long‐term perspective is of particular value, as it captures environmental and hydrological change at centennial and millennial scales (Salgado et al., 2018, 2019; Salgado, Sayer, Brooks, Davidson, Goldsmith, et al., 2018) and supplies limnological information from a scarcely monitored region. This approach has shown to provide continuous data on sedimentological changes and aquatic communities over time allowing to track back in time the effects of land‐use change and its hydrological and limnological effects (Liu et al., 2012; Salgado et al., 2020; Zeng et al., 2018).

By combining multiproxy (fossil diatoms and sediment geochemistry) paleoecological data with historical climatic records from two floodplain lakes (Barbacoas and San Juana) associated with the Magdalena River, this study aims to provide new information on the long‐term limnological responses of these lake systems to both natural hydrological and human‐induced stressors. We expect that the diatom communities will reflect whether the lakes are being affected by anthropogenic activity directly, reflected in a eutrophic diatom composition as a response to local land‐use changes, or indirectly, reflected in diatom dilution due to increases in sediment yield and a sensitivity to hydroclimate reflected on the lake's species turnover regarding extreme dry and wet events. We further hypothesize that in a more connected lake ecosystem (Barbacoas Lake), climatic variation imposes greater controls over the limnology of the lake, while diatom communities are subject to dispersal assembly mechanisms, resulting in lower species turnover, greater variation in species abundance, and a dilution of the within lake signal (Leibold & Norberg, 2004). In less connected lakes (San Juana), the diatom communities are expected to experience marked species turnover, driven by niche assembly rather than dispersion (Leibold & Norberg, 2004). In this sense, the diatom species turnover in these floodplain lakes is expected to be controlled by local abiotic factors (Rodríguez‐Alcalá et al., 2020).

## 2 METHODS

### 2.1 Study area

Barbacoas Lake (6°44′26″N 74°14′36″W) is located on the western margin of the Magdalena River and is directly connected to it via the Barbacoas creek, which has an approximate length of 6.3 km long (Figure 1). Barbacoas is a shallow lake (average depth = 1.2 m) with a superficial area of 10 km^2^, brown‐stained waters (mean secchi depth = 0.39 ± 0.47 cm), pH of 7.25 ± 0.26, and mean daily surface water temperature of 33.7 ± 0.35°C (Table 1). San Juana Lake is located (6°38′32″N 74°09′24″W) on the eastern margin of the Magdalena River (Figure 1). It has a superficial area of 1.05 km^2^ and is characterized by average water depths of 2.2 m, brown‐stained waters (mean secchi depth = 53 ± 0.55 cm), and a mean daily surface temperature of 30.15 ± 0.93°C (Table 1). The lake is fed by the San Juan River on the south that outflows on the west joining later the Carare River before spilling into the Magdalena River near the town of Bocas del Carare (6°46′48″N 74°06′14″W). The hydrological river distance between the Magdalena and the San Juana Lake is approximately of 18.5 km.

**TABLE 1:** Mean values of physical–chemical parameters measured in situ at the littoral and open water areas of the San Juana Lake and Barbacoas Lake during 2018

Lake	Depth (m)	Secchi depth (m)	Temperature (°C)	pH	Dissolved oxygen/surface (ppm)	Dissolved oxygen/bottom (ppm)
*Barbacoas*						
Littoral	0.62	0.29 ± 0.40	33.6 ± 0.59	7.2 ± 0.34	4.84 ± 0.57	2.81 ± 1.23
In‐lake	1.5	0.49 ± 0.54	33.6 ± 0.35	7.3 ± 0.18	5.11 ± 0.64	2.34 ± 1.45
*San Juana*						
Littoral	2.2	0.57 ± 0.79	29.9 ± 1.18	7.5	—	—
In‐lake	2.4	0.5 ± 0.31	30.4 ± 0.68	7.2	—	—

Both lake areas are comprised by wetlands with relatively minor transformations, and small isolated patches of tropical rainforests embedded in a matrix of pastures for extensive cattle ranching that have been pervasively transformed the broader middle Magdalena River basin during the last decades (Figure 1c). Historical mean NDVI vegetation index for the areas surrounding both lakes shows that between 1984 and 2020 changes in vegetation cover and forest structure are due to land‐use changes and interannual climate variability (https://clim‐engine‐development.appspot.com). The NDVI data series consistently indicate that the Barbacoas Lake has more cleared areas related to cattle ranching and agriculture, while the San Juana Lake is surrounded by denser forests. Interestingly, both areas exhibit similar changes throughout the 1984–2020 interval associated with strong El Niño (low NDVI) and La Niña events (high NDVI). The concomitant behavior of both series suggests that major interannual variations in vegetation cover are more attributable to climate variability than to any other driver.

### 2.2 Core sampling

A single, short sediment core from semi‐littoral areas near the mouth of the outflow of each lake was collected using a wide‐bore (diameter of 10 cm) corer (Aquatic Instruments Inc.). The core from the San Juana Lake (LSAN1) was collected at a water depth of 100 cm (6°38′32″N 74°09′24″W). The core from Barbacoas Lake (LBARB1) was retrieved at a water depth of 90 cm (6°44′26″N 74°14′36″W). Each core was subsampled in the field at 1‐cm intervals. The sediments were then kept refrigerated at the Tropical Palynology and Paleoecology Laboratory, Universidad de Los Andes, for further analyses.

### 2.3 Dating and age‐depth model

The LSAN1 and LBARB1 cores were dated using radionucleotide measurements of ^210^Pb, ^226^Ra, ^137^Cs, and ^241^Am by direct gamma assay (Appleby et al., 2001) in the Environmental Radiometric Facility at University College London, UK. For LSAN1, the top 20 cm of the core was dated and an age model beyond the top 20 cm was fitted, by simulating new ages using the “scam” package in R (Pya & Pya, 2021). The age model followed a shape‐constrained generalized additive model (GAM), with the age‐model spline constrained to be monotonic decreasing (Simpson, 2018a). For LBARB1 core, sediment samples were dated every three centimeters, along the whole length of the core. Because of irregular changes in unsupported ^210^Pb activities (Figure S1), the ^210^Pb chronology could not be resolved using the CIC (constant initial concentration) dating model. Thus, the chronology was corrected using the CRS (constant rate of ^210^Pb supply) dating model (Appleby et al., 2001).

### 2.4 Geochemical analysis

The organic matter (OM) content of each core was measured using the method of loss‐on‐ignition (LOI; Dean, 1974). Sampling resolution for LSAN1 core was at 1 cm for the top 20 cm samples and at every 2 cm for the remaining 30 cm of the core samples. For LBARB1 core, sampling resolution was at 1 cm throughout the core. Shifts in OM content were used as a proxy of flooding (river influence) following Schillereff et al. (2014). During high floods, OM is expected to decline through dilution from a greater deposition of terrigenous sediments associated with the river flow (Rapuc et al., 2019). In turn, OM is expected to increase during dry periods through increased in‐lake primary production and decreased allochthonous input (Schillereff et al., 2014).

Sediment geochemistry was measured using X‐ray fluorescence (XRF) with an Xmet 7500 portable analyzer spectrometer (Oxford Instruments Inc.). Three grams of dry sediment, 1‐cm‐thick sample was analyzed for XRF. A sampling resolution of every 1 cm was used for the top 18 cm sediment samples in both cores and of every 3 cm for the remaining bottom samples of both cores. We obtained two XRF readings (1 min of length) for each sediment sample, and the median value of both readings was used for statistical analysis. The XRF portable analyzer spectrometer was calibrated against certified material prior to analysis (Conrey et al., 2014), and all XRF measurements were run using the Mining method, which detects elements occurring in very low (<0.01 ppm) concentrations (Gasdia‐Cocharne, 2017). The selected sediment samples from LSAN1 and LBARB1 cores were analyzed for iron (Fe), manganese (Mn), titanium (Ti), calcium (Ca), zirconium (Zr) phosphorus (P; only for San Juana Lake). The ratios of these elements (excluding P) were used as proxies for river‐borne and in‐lake processes as follows: grain size Zr/Fe (Davies et al., 2015; Schillereff et al., 2014), detrital input Ti/Ca (Davies et al., 2015; Salgado et al., 2020), and oxygenation of the water column Mn/Fe (Davies et al., 2015). Generally, during low flow periods, rivers deliver comparatively less sediments to lakes, and these are normally fine‐grained silts (Schillereff et al., 2014). During slightly elevated flows, clays and Fe commonly occur, whereas during peaks of high floods, coarse‐grained (Zr) sediments are expected to increase (Schillereff et al., 2014). Ti is an unambiguous indicator of allochthonous coarser inputs from the catchment (Cohen, 2003), while Ca is often associated with in‐lake production (Tjallingii et al., 2007). As such, higher values of Ti/Ca ratio may indicate greater detrital input (Salgado et al., 2020). Variation in Fe and Mn provides information about changing redox conditions (Davies et al., 2015). In a reducing (low oxygen) environment, the solubility of Fe and Mn increases, being Mn more readily affected (Boyle, 2002). An increase in Mn/Fe ratio can thus indicate the onset of aerobic conditions. As so, greater river influences into the lakes were inferred by increases in grain size, sedimentation rates, detrital inputs, and concomitant reductions in OM.

### 2.5 Diatoms

Approximately 0.3 g of dry sediment per sample was used for diatom analyses following Battarbee (1986). Each sample was placed in a beaker with 30 ml of hydrogen peroxide (10%) for approximately 24 hr, or until the reaction stopped. After, 100 ml of distilled water was added to each of the samples and they were left until the water column was clear. Then, 0.6 ml of each sample was placed on a microscope slide and allowed to dry after which it was mounted using Naphrax, and then, 400 diatoms were counted and classified. For LSAN1 core, sampling resolution was every 1 cm in the top 20 cm, and every 4 cm for the remaining of the core, for a total of 27 samples. For LBARB1 core, we used a sampling resolution of 2 cm throughout the core, for a total of 22 samples. The differential lake sampling resolution was due to the differences in sedimentation rates and the temporal resolution we wanted to achieve for the recent decades. The diatom species were identified using Lange‐Bertalot and Metzeltin (2007), Krammer and Lange‐Bertalot (1986, 1991a, 1991b), Lange‐Bertalot and Metzeltin (1998), and the Diatoms of North America database (diatoms.org). Diatoms were then grouped into the following functional groups according to their ecological preference: Aerophil, Benthic, and Planktonic (Table 2). For the Benthic category, ecological preferences related to productive and acidic/dystrophic waters were also included (Viktória et al., 2017). Species of the genus *Eunotia*, *Pinnularia*, *Nitzschia*, *Encyonema*, and *Gomphonema* had very low counts and therefore they were aggregated into a single category according to their respective genus.

**TABLE 2:** Diatom species recorded in the sediment record of the San Juana Lake and Barbacoas Lake

Species	Ecology	Functional group	References	Ecological interpretation of diatoms in our study^a^
*Aulacoseira ambigua*	Preference for productive waters, water mixing, and low light conditions.	Plankton	Bicudo et al. (2016)	Mixing conditions, increased turbidity
*Aulacoseira alpigena*	Mixing of the water column, adapted to low light conditions and low pH. Also associated with an increase in runoff. Found in 50 cm of water in the waters from a Paramo cushion mire in Colombia (Velez, personal observation)	Plankton	Bradbury and Van Metre (1997)	Mixing conditions, increased turbidity
*Aulacoseira granulata*	Riverine species commonly found on floodplain lakes. Common in flooding areas (floodplains); reported on similar floodplains in Ayapel, Colombia	Plankton	Hernández‐Atilano et al. (2008)	River influence/mixing conditions
*Aulacoseira granulata var. angustissima*	Eutrophic lakes and rivers	Plankton	Bicudo et al. (2016)	River influence/mixing conditions
*Aulacoseira herzogii*	Mesotrophic to eutrophic lakes; slightly acidic waters	Plankton	Bicudo et al. (2016) Vélez et al. (2005)	River influence/mixing conditions/higher turbidity nutrients
*Aulacoseira distans*	Turbid freshwater. Alkaline and eutrophic ecosystems	Plankton	Tuji (2015)	River influence/mixing conditions
*Cyclotella menenghiana*	Shallow, nutrient‐rich waters	Plankton	Lowe and Kheiri (2015)	River influence/higher turbidity
*Fragilaria*	Mesotrophic to eutrophic lakes	Plankton	Ekdahl et al. (2004)	
*Diadesmis confervacea*	Aerophil, Shallow still water	Aerophil	Raupp et al. (2009)	In‐Lake conditions
*Staurosira pinnata*	Shallow water and high‐quality indicator, high tolerance to dissolved inorganic carbon	Aerophil	Bona et al. (2007)	Shallow waters
*Luticola mutica*	Moses, stones, wet walls, and exposed soil	Aerophil	Liu et al. (2017)	Shallow waters/exposed soil
*Encyonema minutum*	Freshwater species commonly found on weakly acidic environments	Benthic (dystrophic)	Bishop et al. (2017)	In‐lake/dystrophic
*Eunotia*	Low pH conditions	Benthic (dystrophic)	Hée and Gaiser (2012)	In‐lake/dystrophic
*Gomphonema augur*	Lakes with moderately acidic pH; reported on the Amazon floodplains	Benthic (dystrophic)	Lange‐Bertalot and Metzeltin (1998)	
*Neidium sacoense*	Low pH conditions; abundant in wetlands	Benthic (dystrophic)	Burge et al. (2017)	Shallow waters/marsh
*Pinnularia*	Low pH conditions; lakes and wet soil	Benthic (dystrophic)	Hée and Gaiser (2012)	In‐lake/dystrophic
*Placoneis cf.tersa*	Shallow, low alkalinity, and meso‐eutrophic lakes	Benthic (dystrophic)	Poulíčková et al. (2008)	
*Sellaphora alastos*	Ponds and small lakes, oligo‐dystrophic environments	Benthic (dystrophic)	Bahls (2014)	
*Sellaphora laevissima*	Lakes and rivers, mildly acidic environments	Benthic (dystrophic)	Burge et al. (2017)	
*Stauroneis fluminopsis*	Lakes and wetlands	Benthic (dystrophic)	Bahls (2010)	
*Stauroneis neohyalina*	Preference for small humic‐rich lakes and wetlands.	Benthic (dystrophic)	Cantonati et al. (2017)	
*Frustulia crassinervia*	Oligotrophic habitats	Benthic (dystrophic)	Kulichová and Fialová (2016)	
*Gomphoneis eriense*	Found on lakes; sensitive to human disturbance; tolerant to turbulence	Benthic (productive)	Kociolek and Stoermer (1988)	
*Nitzschia*	Tolerant to pollution and high dissolved carbon	Benthic (productive)	Ramirez and Plata‐Diaz (2008)	
*Actinella disjucta*	Environments rich in humic acids. Bog flora	Benthic (productive)	Lange‐Bertalot and Metzeltin (2007)	Productivity/river influence
*Hantzschia elongata*	Ponds and wetlands, eutrophications	Benthic (productive)	Loganathan et al. (2014)	

Note

The ecology, associated functional group, reference, and ecological preference suggested by our data is presented.

^a^ The ecological interpretations of the diatoms were assessed only for the most relevant species observed in the paleoenvironmental reconstruction of the lakes.

### 2.6 Data analysis

#### 2.6.1 Changes in diatom assemblages

To detect major zones of temporal diatom and geochemical change, we used stratigraphically constrained hierarchical clustering (Coniss) analysis on Bray–Curtis dissimilarities using the *Rioja* package in R (Juggins, 2009. The main compositional changes in the diatom communities were then assessed via Rank clocks analysis (RCA; Collins et al., 2008) using the *Codyn* package in R (Hallett et al., 2016). RCA is a useful technique for visualizing species reordering through time as the analysis plots species according to the rank order of abundance on a clock‐like diagram, where 12 o'clock on the vertical axis is the starting point of the data, where the oldest sediment samples would be; time moves clockwise and the top of the core is plotted at the left of 12 o'clock as it comes full circle (Collins et al., 2000). Variations in rank order of species abundances are shown according to proximity of the center of the diagram. Abundant species at a particular time period are for instance, plotted away from the center of the diagram, while species having lower abundances are plotted in proximity to the center of the diagram. Prior to Coniss and RCA analyses, diatom counts were square‐root‐transformed in order to weight the varying relative abundances of the different diatom species (Oksanen et al., 2010).

#### 2.6.2 Hydroclimatic variation and lake responses

To quantify how lakes have responded to extreme long‐term climatic events (ENSO), we run a Standardised Precipitation‐Evapotranspiration Index–SPEI analysis (Vicente‐Serrano et al., 2010). This analysis uses historical climatic data to generate a drought index based on the difference between precipitation and potential evapotranspiration across a given area, allowing the identification of years with extreme drought or excess water (Vicente‐Serrano et al., 2010). The SPEI index data were downloaded from https://spei.csic.es/map/maps.html#months=1#month=3#year=2020 for the interval between 1985 and 2016. Years with severe drought or excess precipitation were obtained using the annual mean data (i.e., 12‐month time scale). The Global SPEI database is fed by worldwide monthly drought conditions data with a spatial resolution is of 0.5° (Vicente‐Serrano et al., 2010).

Years with SPEI index values between −0.5 and 0.5 are considered to fall within normal conditions (Vicente‐Serrano et al., 2010), whereas years with values <2 are considered as extremely wet, and values >−2 are considered as extremely dry (Vicente‐Serrano et al., 2010). Extreme wet and dry years were identified and contrasted against the surface area of the lakes during that specific year. The total surface area of each lake during these events was calculated through polygons in a supervised image classification analysis in Qgis desktop 3.10.5.

#### 2.6.3 Gradients of ecological, geochemical and climatic change

The main temporal gradients of ecological, geochemical, and climatic change for each lake were assessed using multiple factor analysis–MFA (Pagès, 2002). MFA allows to cluster the different geochemical parameters (element ratios, LOI, and sedimentation rates), diatom functional groups (acidic/dystrophic, aerophil, planktonic, and benthic/productive), and the SPEI annual data (mean, maximum and minimum) into specific groups and assess simultaneously the amount of variation explained by each group. Trends in trajectory of change in the multidimensional space can be also visually assessed. The geochemical, diatom, and SPEI groups were standardized by applying a weight equal to the inverse of the first eigenvalue of the analysis of the group (Pagès, 2002). The MFAs were performed in R using the package *FactoMineR* (Pagès, 2002).

#### 2.6.4 Significant periods of change

Generalized additive models (“mgcv” package in R, Wood & Wood, 2016) were then used to estimate significant trends of temporal change using smooth functions following Simpson (2018a). The GAMs were fitted to the MFA DIM 1 scores of each lake against time (Beck et al. (2019) and the residual maximum‐likelihood (REML) method was used to penalize overfitting trends. A Gaussian distribution with an identity link was used to model the time series data, and diagnostic Q–Q plots were performed to check for homogeneity of variances in the residuals. To account for uneven observations in the time series and for age uncertainty (heteroscedasticity) in the models, we used the amount of time per sediment sample/divided it by its means as a weight following Simpson (2018a). A base function (*k*) of 15 was used to achieve the best model fit (see Table S1 in Appendix for more details). The first derivative function of each GAM was identified and used to determine significant trends in the time series data using the “gratia” package in R (Simpson, 2018b). Here, trends that deviated from 0 (no trend) indicated periods of significance (Simpson, 2018). The strength of nonlinearity in the driver response relationships was also assessed using the effective degrees of freedom (edf) of the GAMs (Hunsicker et al., 2016). An edf equal to 1 is equivalent to a linear relationship, whereas an edf >2 implies a highly nonlinear relationship and thus most likely to exhibit ecosystem threshold responses (Hunsicker et al., 2016).

## 3 RESULTS

### 3.1 Age model and sedimentation rates

**FIGURE 2:**
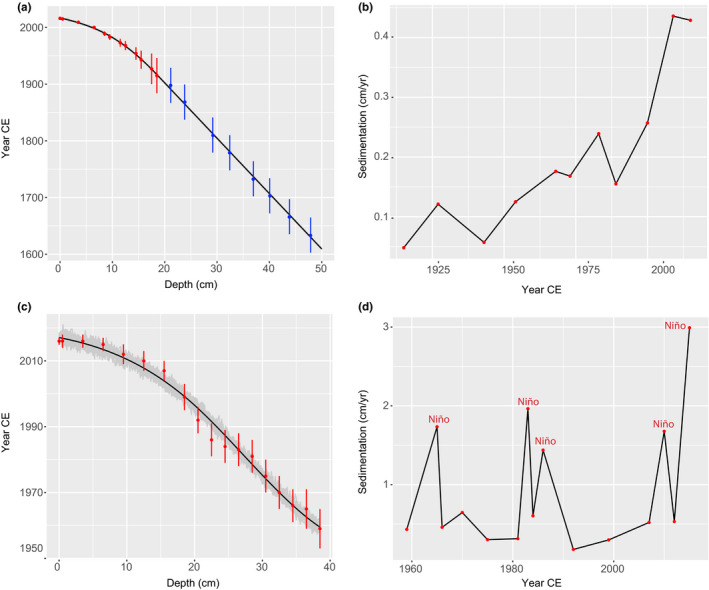
(a) Radiometric chronology of the core LSAN1 (San Juana Lake) showing the age model, ^210^Pb dates are indicated by red dots and predicted dates bellow the ^210^Pb dates are indicated by blue dots; (b) sedimentation rates in LSAN1; (c) radiometric chronology of the core LBARB1 (Barbacoas Lake) showing the age model, ^210^Pb dates are indicated by red dots; (d) sedimentation rates in LBARB1 core

The LSAN1 core was of 50 cm long with ^137^Cs and ^241^Am activities indicating the 1963 maximum fallout of the atmospheric nuclear bomb around the top 19‐cm section (Figure S1a). The resulting ^210^Pb age model indicates that the top 19 cm covered the last 100 years with the predicted dates bellow this core depth suggests a sediment record covering approximately to 1622 CE (Figure 2a). Sedimentation rates within the ^210^Pb dated portion of the core (top 19 cm) range from 0.048 to 0.436 cm/year (Figure 2b). Pre‐1950s, rates remain relatively stable fluctuating between 0.048 and 1.12 cm/year (mean = 0.088). Between 1968 and 1989, sedimentation rates increased by around onefold varying between 0.15 and 2.4 cm/year (mean = 0.18). Post‐2000s, sedimentation rates gradually doubled reaching a current maximum of 0.43 cm/year.

The L‐BARB1 core was 40 cm long with the corrected CRS aged model putting 1963 depth at around 36.5 cm (Figure 2c). Very low ^210^Pb activities within the top first centimeters of the core indicate possible sediment mixing (Figure S1b), and thus, the top 3.5 cm sediments were assumed to be formed within the same year. Sedimentation rates ranged between 0.43 and 2.99 cm/year with four marked peaks at 1965 (1.73 cm/year), 1983 (1.96 cm/year), 1987 (1.43 cm/year), and 2010 (1.67 cm/year) (Figure 2d). Post‐2010, sedimentation rates spiked to 2.99 cm/year.

### 3.2 Diatoms

#### 3.2.1 San Juana Lake

**FIGURE 3:**
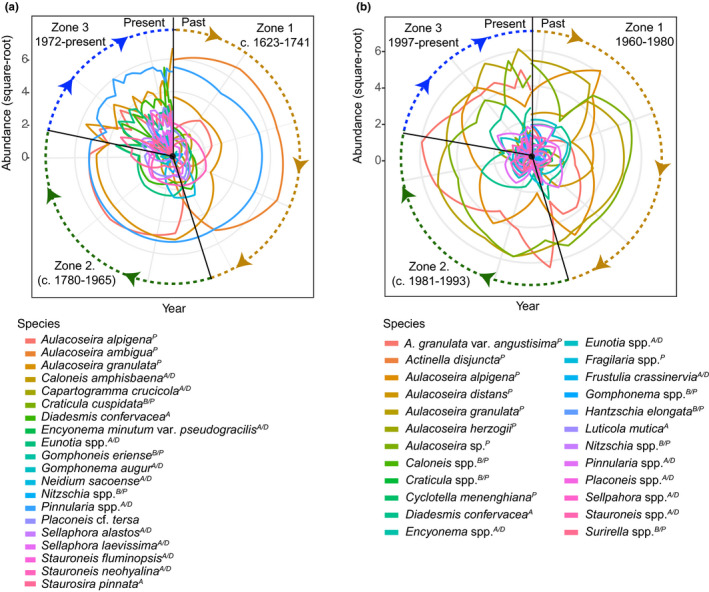
Results of RCA on diatoms assemblages of (a) LSAN1 core and (b) LBARB1 core. RCA identifies which species show the greatest change in abundance (i.e., those distancing from the center of the diagram) at temporal zones of change revealed by clustering analysis on a clock‐like diagram, where 12 o'clock on the vertical axis is the starting point of the data. Major zones of diatom community change in both lakes detected by coniss analysis are indicated with brown (*Zone 1*), green (*Zone 2*), and blue (*Zone 3*). A/D = acidic/dystrophic; B/P = benthic productive; P = planktonic; A = aerophil

A total of 19 diatom taxa were found in LSAN1 (Figures 3a and S4). Clustering analysis and RCA revealed the following major zones of change in the diatom assemblages:

##### Zone 1 (48.5–36.5 cm; c. 1622–1747 CE)

This zone was characterized by dominance of *Aulacoseira ambigua* (Figure 3a). The diatoms *Pinnularia* spp. *Aulacoseira granulata*, *Neidium sacoense*, *Capartogramma crucicola*, and *Caloneis amphisbaena* also occurred but with lower abundances.

##### Zone 2 (35.5–12.5 cm; c. 1758–1964 CE)

This zone was characterized by a codominance of *Pinnularia* spp., *A. granulata*, and *A. alpigena* (Figure 3a). The species *Diadesmis confervacea* and *Staurosirella pinnata* were also present in low abundances.

##### Zone 3 (22.5–0.5 cm; c. 1967–2016 CE)

This zone was dominated by *Pinnularia* spp. and *A*. *granulata* (Figure 3a). The diatom species *A. alpigena* was present but with lower abundances compared to the previous zone. *D. confervacea*, *Eunotia* spp., and *Sellaphora alastos* increased while other diatoms present, but in lower abundances included *Stauroneis neohyalina*, *Gomphoneis eriense*, and *Encyonema minutum* var. *pseudogracilis*.

#### 3.2.2 Barbacoas Lake

A total of 21 diatom taxa were found in LBARB1 core (Figures 3b and S5). Clustering analysis and RCA indicated three main temporal zones of diatom assemblage change:

##### Zone 1 (38.5–24.5 cm; c. 1959–1984 CE)

In this zone *A. granulata*, *Aulacoseira* sp., *A. alpigena,* and *Actinella disjuncta* dominated the assemblages (Figure 3b). In minor proportions and mainly restricted to this zone were *A. distans* and *D. confervacea*. *A. herzogii* peaked around 22 cm. Other taxa occurring with lower abundances included *Eunotia* spp., *Pinnularia* spp., and *Cyclotella meneghiniana*.

##### Zone 2 (29–22 cm; c. 1989–1992 CE)

During this time, *A. granulata, A. granulata* var. *angustissima, Aulacoseira* sp., *A. alpigena* and *D. confervacea* dominated (Figure S5). It is important to notice that between 21 and 22 cm (1989 and 1992, respectively) species such as *A. herzogii* and *C. meneghiniana* disappeared from the fossil record but reappeared post‐1992. Also, *Luticola mutica* appeared around 22 cm (1989) peaking around 21 cm (1992). *Pinnularia* spp., *Encyonema* spp., and *Eunotia* spp. were present in lower proportions and remained constant through this interval.

##### Zone 3 (21–0 cm; c. 1989–1992 CE)

This zone was marked by dominance of *A. granulata* var. *angustissima* along with increases in *A. herzogii*, *Frustulia crassinervia*, and *Pinnularia* spp. (Figure S5). At the core depths 15 and 5 cm (2011 and 2015, respectively), all diatoms disappeared from the record.

### 3.3 Geochemical analysis

#### 3.3.1 San Juana Lake

LOI values in LSAN1 core ranged between 8.9% and 14.5% between *c*. 1622 and 1927 CE (Figure S2a). A subsequent gradual increase was observed being more pronounced after 1995 with current LOI values around 29%. The Mn/Fe (oxygenation of the water column) values were also low between *c*. 1622 and c. 1858 CE ranging between 0.0011 and 0.0021 ppm. A gradual increase with a more pronounced increase was observed after 1994. Modern Mn/Fe values almost doubled historical values. The Ti/Ca (detrital inputs) and Zr/Fe (grain size) ratios were both generally high between c. 1622 and 1917 CE (ranging between 1.33 and 0.03 ppm, respectively) but gradually declined afterward up to half the historical values. P concentrations in the lake sediments also showed a gradual increase from historical values ranging around 0.17 and 0.12 ppm between c.1622 and 1917 CE to current values of 0.27 ppm.

#### 3.3.2 Barbacoas Lake

LOI values in LBARB1 core were relatively high between 1960 and 1987, ranging between 26% and 22% (Figure S2b). A marked subsequent decrease in LOI values was observed since; current values were around 9.3%. The Mn/Fe ratio fluctuated over time ranging between 0.0008 and 0.0012 with a slight tendency of increasing toward recent times. The Ti/Ca showed a gradual decrease between 1960 and 1980 from 0.71 to 0.39 ppm, followed by a gradual recovery phase toward recent times. The Zr/Fe ratio was relatively low (0.0001) between c. 1960 and 1987 CE. A subsequent pronounced increase was observed, where current values were around threefold higher than those historically observed.

### 3.4 SPEI analysis

**FIGURE 4:**
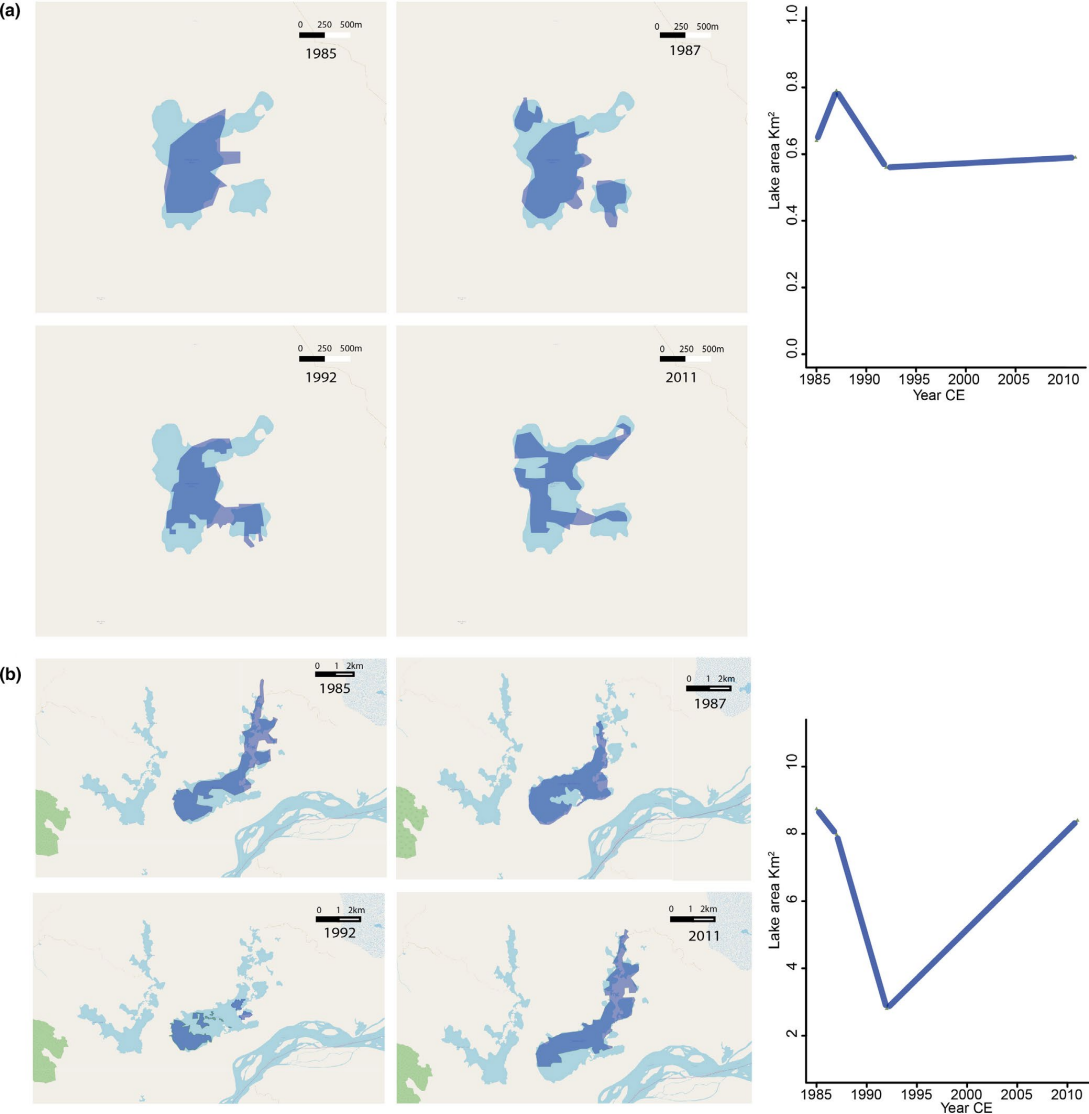
Variations in lake surface area (km^2^) of the San Juana (a) and Barbacoas (b) Lakes during 1985 (Barbacoas Lake 8.7 km^2^; San Juana Lake 0.64 km^2^), 1987 (Barbacoas Lake 7.98 km^2^; San Juana Lake 0.79 km^2^), 1992 (Barbacoas Lake 2.8 km^2^; San Juana Lake 0.56 km^2^), and 2011 (Barbacoas Lake 8.4 km^2^; San Juana Lake 0.59 km^2^)

Extreme dry and wet periods (Figure S3) were identified: 1985 (was classified as reference/neutral), 1987, and 1992 were extremely dry, while 2011 was wet. Calculated lake surface area values (Figure 4) for San Jana were 0.64 km^2^ in 1985, 0.79 km^2^ in 1987, 0.56 km^2^ in 1992, and 0.59 km^2^ in 2011 (Figure 4a). Lake surface area for Barbacoas was of 8.7 km^2^ in 1985, 7.98 km^2^ in 1987, 2.8 km^2^ in 1992, and 2011 8.4 km^2^ in 2011 (Figure 4b).

### 3.5 Gradients of ecological, geochemical, and climatic change

**FIGURE 5:**
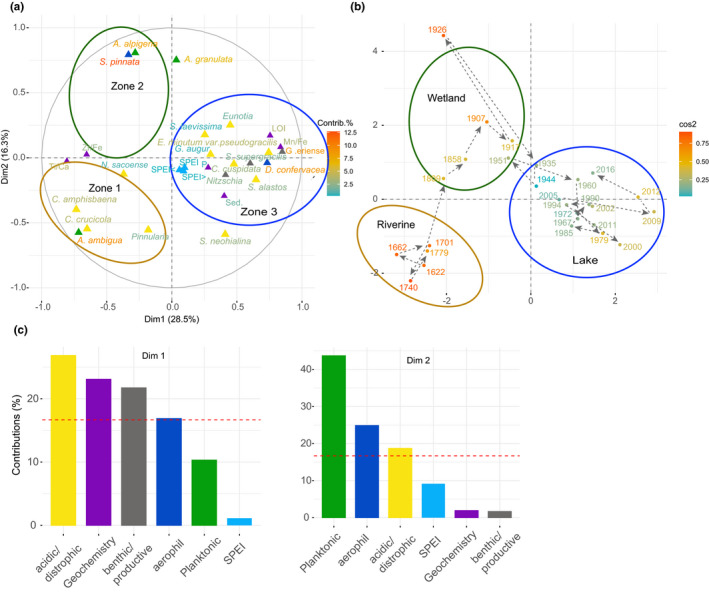
Multiple factor analysis (MFA) plots for the core LSAN1, showing (a) the variation and contribution of diatoms functional groups (plankton in green, acidic/dystrophic in yellow and benthic/productive in gray), selected geochemical ratios and elements (purple) Fe/Mn, Ti/Ca, Zr/Fe, and P, organic matter content (LOI) and sedimentation rates and SPEI data (SPEI = annual average; < minimum annual value; > maximum annual value). Major zones of diatom community change detected by coniss analysis are indicated with brown (*Zone 1*), green (*Zone 2*), and blue (*Zone 3*); (b) trajectory of change (black dashed arrows) in sediment samples across the multivariate space; (c) contribution of diatom functional groups, geochemistry, and SPEI related to MFA dimensions 1 and 2. For (a) and (b) the contribution of each variable/sampling point is indicated according to a color scale, being red the highest value and green the lowest

The MFA for San Juana Lake showed that Dimension 1 (Dim 1) explained 28.5% of the total diatom, geochemical and climatic variation, whereas dimension 2 (Dim 2) explained 16.3% (Figure 5). Acidic/dystrophic diatoms (27%), geochemical processes (24%), benthic/productive (23%), and aerophil diatoms species (17%) explained most of the Dim 1 variation. Planktonic (44%), aerophil (25%), and acidic/dystrophic (19%) diatom species explained most of the variation of Dim 2. Dim 1 had a strong negative association with the planktonic diatoms *A. ambigua* and the acidic/dystrophic *Pinnularia* spp., *C. amphisbaena*, *C. crucicola*, and *N. sacoense*. Detrital inputs (Ti/Ca) and grain size (Zr/Fe) were also negatively correlated with Dim 1. The aerophil *D. confervacea*, the benthic/productive *Nitzschia* spp., *G. eriense*, and *C. cuspidata*, and the acidic/dystrophic, *Eunotia* spp., *S. alastos*, and *S. supergracilis* were all positively related to Dim 1, along with LOI, Mn/Fe, and sedimentation rates. A positive correlation with Dim 2 was observed with the planktonic *A. alpigena*, and *A. granulata* and the aerophil *S. pinnata*. Sediment samples moved over time along the MFA diagram accordingly, with Zone 1 (c. 1600s–1779 CE) samples placed on the lower left‐hand side of the diagram. Zone 2 (c. 1758–1964 CE) samples moved toward the top center of the diagram. Samples moved then toward the right‐hand side of the multivariate diagram forming Zone 3 (1961–2016 CE).

**FIGURE 6:**
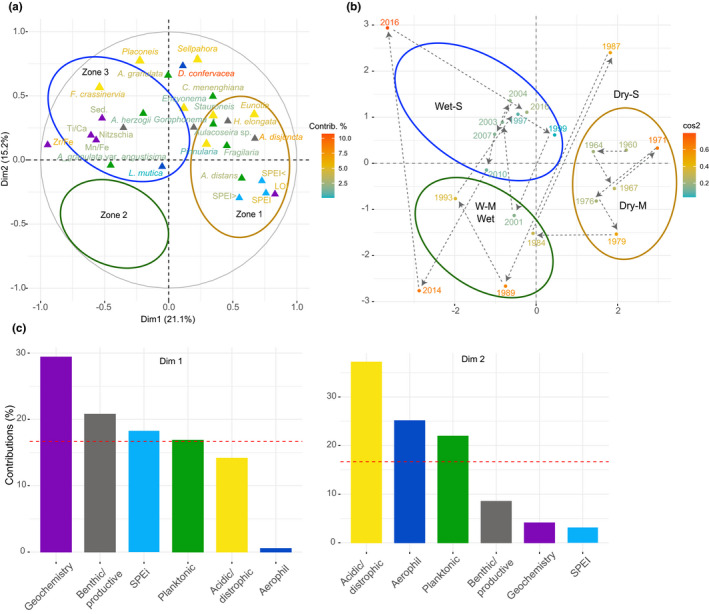
Multiple factor analysis (MFA) plots for the core LBARB1, showing (a) the variation and contribution of diatoms functional groups (plankton in green, acidic/dystrophic in yellow, and benthic/productive in gray), selected geochemical ratios and elements (purple) Fe/Mn, Ti/Ca, Zr/Fe, and P, organic matter content (LOI), and sedimentation rates and SPEI data (SPEI = annual average; < minimum annual value; > maximum annual value). Major zones of diatom community change detected by coniss analysis are indicated with brown (*Zone 1*), green (*Zone 2*), and blue (*Zone 3*); (b) trajectory of change (black dashed arrows) in sediment samples across the multivariate space; (c) contribution of diatom functional groups, geochemistry and SPEI related to MFA dimensions 1 and 2. For (a) and (b) the contribution of each variable/sampling point is indicated according to a color scale, being red the highest value and green the lowest

The MFA for Barbacoas Lake showed that Dim 1 explained 21.1% of the total diatom, geochemical and climatic variation, while Dim 2 explained 15.2% (Figure 6). The main groups characterizing DIM1 were geochemistry (29%), benthic/productive diatoms (22%), SPEI (18%), and planktonic diatoms (17%). Acidic/dystrophic (36%), aerophil (25%), and planktonic (22%) diatoms contributed the most to Dim 2 variation. Dim 1 had a strong positive association with the SPEI data and LOI. Diatom species also positively related to DIim 1 the planktonic *A. distans*, *Fragilaria* spp., and *A. alpigena*, the benthic/productive *A. disjuncta* and *Hantzchia elongata* and the acidic *Eunotia* spp. A strong negative association with D1 was also observed for the geochemical ratios Zr/Fe, Ti/Ca, Mn/Fe, and sedimentation rates. The planktonic *A. herzogii*, and *A. granulata* var. *angustisima*, the benthic/productive *Nitzchia* spp., the acidic/dystrophic *Encyonema* spp., *Stauroneis* spp., and *F. crassinervia* and the aerophil *L. mutica* were also negatively related to Dim 1. A strong positive association between Dim 2 and the acidic/dystrophic *Sellaphora* spp., Placoneis spp., and *Encyonema* spp., the planktonic *C. menenghiniana,* and the aerophil *D. confervacea* was also observed. Sediment samples moved over time along the MFA diagram accordingly, with Zone 1 (1960–1980 CE) samples placed on were placed on the right‐hand side of the plot. Zone 2 (c. 1984–2001) samples moved toward the left‐downside of the diagram. Samples moved subsequently toward the top‐left side of the multivariate diagram forming Zone 3 (1999–2016 CE).

### 3.6 GAMs and derivatives

**FIGURE 7:**
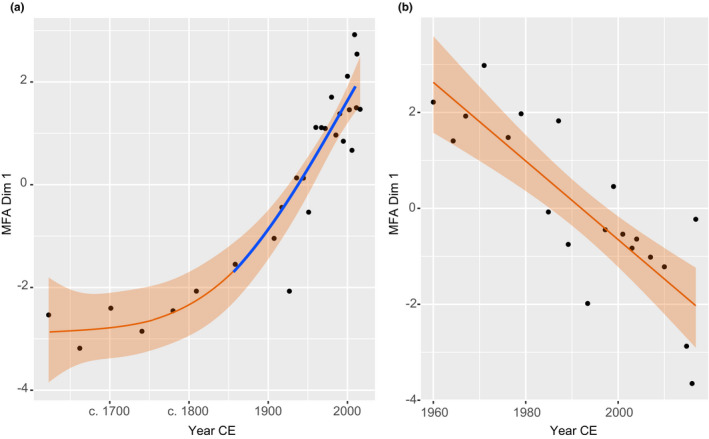
Generalized additive model plots on temporal changes in MFA dimension 1 in the cores (a) LSAN1San—Juana Lake; and (b) LBARB1–Barbacoas Lake. Significant periods of change detected by first derivate analysis are shown in blue

The statistical results for each lake GAM fits can be found in the Supplementary Data (Table S1). For San Juana Lake, the edf value was >2 indicating a nonlinear behavior (Figure 7). The first derivate results detected a significant threshold after c. 1858 that gradually continued up to the present. The analysis for Barbacoas resulted in an edf value of 1 indicating a strong lineal response over time with no significant thresholds.

## 4 DISCUSSION

Our results were in general agreement with previous riverine metacommunity studies (Eros et al., 2012; González‐Trujillo et al., 2020; Grant et al., 2007) and support our initial hypothesize that in a more connected lake ecosystem, climate and dispersal‐related assembly mechanisms are more important than at the less connected lake, where niche assembly rather than dispersion and climate results in marked species turnover. For instance, since the establishment of modern conditions, the more isolated San Juana Lake has been less dependent on the hydroclimatic variations and thus less vulnerable to interannual climate variability. Moreover, the post‐1980 extreme dry and wet events did not greatly affect the lake's surface area or its physical–chemical conditions. However, the marked temporal successional trend in diatom species and the more recent (post‐1960s) increases in diatom species associated with productive environments (*Nitzschia* spp., *C. cuspidata* and *G*. *eriense*; Table 2), coupled with gradual increases in P, OM, and sedimentation rates, all indicate greater importance of in‐lake factors in driving the diatom communities. The more enclosed nature of the San Juana Lake may therefore facilitate a quicker response to localized stressors such as deforestation, and nutrient runoff from agriculture and husbandry (Bennion et al., 2004; Salgado et al., 2019).

In contrast, we found that Barbacoas Lake is prone to be highly sensitive to hydroclimatic events such as ENSO, where changes in diatom dominance are far more important than turnover. The geochemistry of the lake also strongly correlates with ENSO. We found greater OM production occurring during dry periods, while greater detrital inputs during wet periods. Spikes in sedimentation rates were also found to correlate with dry ENSO periods (Figure 2), likely associated with the first coming rains of the wet season. Nonetheless, the increasing tendency of sedimentation rates post‐2000 suggests that other factors rather than climate are also influencing sediment deposition in the lake. A recent study by Restrepo and Escobar (2018) similarly found that after accounting for the effects of precipitation, sedimentation rates have severely augmented since 2000s in the Magdalena River in response to the increasing forest clearance in the river catchment.

Ecosystem regime shifts are usually characterized by a gradual environmental change which eventually pushes an ecosystem to a critical threshold (Dakos et al., 2015). Determining the existence and nature of such ecological changes is, therefore, key for floodplain conservation and for understanding ecosystem resilience (Dakos et al., 2015). As shown here, regime shifts are challenging to identify as they could be slow and derived from multiple natural and human‐derived causes (Bunting et al., 2016). More importantly, the rate of ecosystem response to environmental change could also depend on the degree of hydrological connectivity (Salgado et al., 2019). We show that connected lake systems may respond slowly in a gradual lineal fashion but with high variance determined by ENSO. In turn, isolated lakes may be prone to respond faster but in a nonlineal way with low variance. These results open a window for tropical floodplain lake landscapes as model systems to test ecological theory of long‐term ecosystem regime shifts controlled by multi‐annual hydrological connectivity.

### 4.1 Long‐term evolution of lakes and their response to extreme events

#### 4.1.1 San Juana Lake

The San Juana Lake transitioned over time from a river‐governed system, to a wetland, and eventually to the lake it is today, probably as a consequence of a progressive disconnection from the Carare River, tributary of the Magdalena River (Figure 1). This is interpreted from the dominance in planktonic diatom species such as *A. ambigua* and *A. granulata*, and *C. amphisbaena* around the early c. 1600s and late 1700s (Zone 1) that reflect water mixing and river influence (Table 2). This is in accordance with the geochemical data that indicate low concentration of OM, high detrital inputs, and grain size. After this period, our analysis identified a significant ecosystem threshold at around the mid‐1800s (Figure 7), linked to an isolated wetland‐like system transition (Zone 2). This limnological change was indicated by decreases in the riverine–planktonic *A. ambigua* along with a marked increase in *A. alpigena*, a species associated with low waters levels in tropical freshwater lakes (Table 2). The prevalence of *S. pinnata* and the presence of the aerophil *D. confervacea* further support a very shallow, wetland‐like environment (Table 2). Accordingly, the declines in grain size and detrital inputs further suggest a disconnection to the river (Salgado et al., 2020; Schillereff et al., 2014). Such long‐term transition from river, to a wetland‐like environment, fits other ontological process of South American floodplain lakes (Fayó et al., 2018). These commonly originate from a natural cutting off the meandering neck of a river (Gaiser & Rühland, 2010), likely assisted by pronounced shifts in precipitation regimes and sediment load that alter connectivity (Amoros & Bornette, 2002). As shown here, ENSO can bring extreme dry periods in the study area; hence, the change from a river‐dominated system to a wetland‐like environment would have been likely derived from a historical strong ENSO event (Li et al., 2011). Such dry climatic conditions would have promoted a disconnection of a meander from the main river channel (Fayó et al., 2018).

Permanent modern lake conditions were established around the late 1960s, with moderately water acidic conditions (Zone 3), as indicated by the dominance of benthic/tychoplanktonic rather than planktonic diatoms including *Stauroneis neohyalina*, *Sellaphora alastos*, *Gomphoneis eriense*, and *Encyonema minutum* var. *pseudogracilis* (Table 2). The transition to a lake system is also supported by further, and more pronounced, declines in detrital inputs and grain size along with an increase in autochthonous productivity (OM content) and water column oxygenation. Replacements of coarser sediment beds, to finer sands and clays in response to a river–lake transition, have been similarly recorded by Kuerten et al. (2013) in the Pantanal floodplains, in Brazil. Similarly, increases in OM and water column oxygenation after artificial damming, or as in our case, after the fluvial input declined, have been also described after the Panama Canal construction (Salgado et al., 2020) and in river‐dammed floodplain lakes in the Yangtze River, China (Liu et al., 2012; Zeng et al., 2018).

#### 4.1.2 Barbacoas Lake

The sediment record of Barbacoas Lake showed that the system has been responding to a varying climatic influence and associated degree of connectivity to the main river but without reaching any ecosystem threshold (as indicated by the lineal response in GAM; Figure 7). Between 1960 and 1984 CE, the association between high SPEI values (>0) and high OM content coupled with the dominance of *A. distans*, *A. alpigena*, *Pinnularia* spp.*, Eunotia* spp., and *C. meneghiniana* (Figure 6) indicates a relatively dryer climate and wetland‐shallow, productive, and turbid water conditions (Table 2; Fayó et al., 2018). Such macrophyte‐rich wetland areas would have also provided suitable habitats for the detected dominant aerophil *D. confervacea* during the strong El Niño period of 1987 (Figure 6).

Post‐1985, extreme drier and wetter conditions further influenced the limnology of Barbacoas Lake. For instance, the strong droughts caused by El Niño of 1987 and 1992 resulted in severe reductions of the lake surface water area (Figure 4); events that also caused the partial desiccation of most shallow waterbodies in Colombia (Pestana Calderín & Mejía Arroyo, 2011). Such drier conditions not only would have diminished the lake–river connectivity, promoting disconnection from the Magdalena River, but also must have increased evaporation rates resulting in the partial desiccation of littoral areas, a process that was observed during a subsequent dry spell in 2020 across the lakes in the study area (personal observation by Lopera, L & Salgado, J). In such smaller surface lake area with greater exposed littoral areas, aerophil species such as *L. mutica* and *D. confervacea* (Table 2) would have again found ideal opportunities to thrive (Figure 6).

In a similar fashion, the direct connection to the Magdalena River was suggested to be re‐established and enhanced during extreme and moderate wet periods, where the lake recovered its original surface area (Figure 4). In 2011, for instance the country experienced one of the greatest La Niña events of the last century, where most of the Magdalena River catchment experienced unforeseen floods (Euscategui & Hurtado, 2011). Our record shows that, at the time, diatom counts were barely legible in the fossil record while sedimentation rates increased significantly. Such large increases in river sediment inputs would have affected diatom preservation and/or dilute the diatom concentrations (Reed, 1998; Salgado et al., 2020). We also found that during moderate wet periods, grain size, water column oxygenation, and detrital inputs all increase in the lake. Diatoms species such as the planktonic *A. herzogii*, *A. granulata* var. *angustissima,* and *A. granulata* and the benthic *Nitzschia* spp. also increased in abundances during these wet periods.

### 4.2 Paleoflood interpretation

Overall, we were able to discern the level of connectivity and river influence on the lakes through our multiproxy approach. For instance, we found a good concordance between dry years (SPEI values < 0), and high OM content and low grain size ratio (Schillereff et al., 2014). It is expected that with a lower river influence, the OM in the systems increases through in‐lake primary productivity (Schillereff et al., 2014). As seen in Barbacoas Lake, the increasing river influence is thus reflected by a decrease in OM being deposited by the river and by an increase in detrital inputs (Rapuc et al., 2019), an opposite trend to what was observed in the San Juana Lake since its formation. The prevailing benthic/productive diatom functional group associated with these dry periods also concurred with previous floodplain lake literature (Fayó et al., 2018; Gell & Reid, 2014; Liu et al., 2017). Similarly, we found lower OM content and high grain size and detrital inputs to be associated with wet (SPEI < 0) periods (Rapuc et al., 2019; Schillereff et al., 2014). Planktonic diatom species also prevail during wet periods, in particular *A. granulata* var. *angustissima* and *A. herzogii* (Fayó et al., 2018; Gell & Reid, 2014; Liu et al., 2017). These results provide therefore an exciting tool to assess with confidence lacking long‐term monitoring data on hydrological and ecological change in the Magdalena River system.

### 4.3 Limitations

Paleoecological data have limitations and can be biased. For instance, uncertainties in the age model of San Juana Lake and potential sediment reworking by waves and bioturbation may have introduced discrepancies in our age models. In particular, the simulated ages (pre‐1900s) beyond the ^210^Pb dated portion in the San Juana core must be understood as modeled and taken with caution. Similarly, as floodplain lakes combine riverine and lacustrine features, limnological processes may have large spatial variations across the lakes that may have been not fully accounted by our single‐core approach (Maavara et al., 2015). Nonetheless, our multiple and independent lines of evidence of change in biotic and abiotic variables are all in general agreement. The magnitude and timing of changes observed in our record coincide with the known basin‐wide natural climatic and anthropogenic history of the Magdalena River (e.g., Restrepo & Escobar, 2018) as well as with other tropical floodplain lake system dynamics in general (Kuerten et al., 2013; Liu et al., 2012; Zeng et al., 2018). The wide range of proxies used in our study also represents a different geographical extent. For example, geochemical elements integrate basin‐wide information (Davies et al., 2015), while diatoms represent what is occurring in the lake scale (Pla‐Rabés & Catalan, 2018); thus, the combination of several proxies would also imply the integration across spatial scales. Being so, the agreement among proxies implies not only a synchronic behavior but also a coincidence among scales. Thus, we are confident that despite our single‐core approach, our data are reflective of a general historical change in each lake.

## 5 CONCLUDING REMARKS

During the last four decades, the Magdalena River basin has witnessed unprecedented transformations in land cover as the middle Magdalena valley is mainly being exploited for gold, cacao, palm oil, petroleum, and cattle farming (Cámara de Comercio de Medellín, 2014; Etter et al., 2006; Restrepo & Escobar, 2018; Suescún et al., 2017). Forest clearance has greatly promoted erosion, increasing sediment loads and nutrients into the Magdalena River (Restrepo & Escobar, 2018). The signal of these marked increases in sedimentation in both lakes suggests that the current tendency of land‐use change and higher erosion should be a factor to consider when assessing future management strategies, as the impacts of this are not yet clear in these lakes.

We also showed that the two studied lakes have had a very different ontological histories controlled by the degree of connectivity to the Magdalena River and climatic variation. The higher degree of connectivity of Barbacoas Lake makes it far more sensitive to ENSO events than the more isolated San Juana Lake, which in turn is suggested to be more sensitive to local changes associated with its watershed. Future climate change scenarios suggest that drier conditions will prevail in our study area (IDEAM, 2017). For the more isolated lakes, this could likely increase water retention times promoting in‐lake productivity and generating cascade effects such as anoxia and eutrophication (Chislock et al., 2013). The degradation of these smaller and more isolated lakes is of great concern. These lakes play key roles in water regulation and in offering temporal refuge for the aquatic biota including endangered large mammals such as the river otter and the American manatee (Wild Conservation Society–WCS Colombia, 2015), and key economic species such as the Magdalena catfish or the Magdalena prochilodontid. As observed in Barbacoas, drier climates will inevitably lead to reductions in lake size, which will diminish habitats and the hydrologic regulatory capacity of these lakes (Amoros & Bornette, 2002). These processes will likely have positive feedbacks as more sediment will be accumulated allowing for emergent plants to colonize newly formed habitats, which in turn, will promote the lake disconnection (Amoros & Bornette, 2002). This, with the added concern of the increased sedimentation rates, will put the lake at risk of rapid clogging, and disappearance.

## CONFLICT OF INTEREST

We declare no conflict of interest regarding patent or stock ownership, membership of a company board of directors, membership of an advisory board or committee for a company, and consultancy for or receipt of speaker's fees from a company.

## AUTHOR CONTRIBUTION

**Laura Lopera‐Congote:** Conceptualization (equal); Data curation (lead); Formal analysis (equal); Investigation (lead); Methodology (lead); Writing‐original draft (lead); Writing‐review & editing (equal). **Jorge Salgado‐Bonnet:** Conceptualization (lead); Formal analysis (supporting); Funding acquisition (equal); Investigation (lead); Methodology (supporting); Project administration (equal); Resources (supporting); Supervision (lead); Validation (equal); Visualization (equal); Writing‐review & editing (equal). **Maria Isabel Vélez:** Conceptualization (equal); Data curation (equal); Formal analysis (equal); Funding acquisition (supporting); Investigation (equal); Methodology (equal); Resources (equal); Supervision (lead); Writing‐review & editing (equal). **Andres Link:** Funding acquisition (equal); Investigation (equal); Methodology (equal); Resources (equal); Writing‐review & editing (equal). **Catalina González:** Funding acquisition (equal); Investigation (equal); Methodology (equal); Resources (equal); Supervision (supporting); Writing‐review & editing (equal).

## Supporting information

Supinfo S1

## Data Availability

The data supporting the results are archived in Pangaea Data Repository (https://doi.pangaea.de/10.1594/PANGAEA.933157; PDI‐28067).
