# Supplementary material for: River connectivity and climate behind the long‐term evolution of tropical American floodplain lakes
Source: Ecol Evol. 2021 Sep 14;11(19):12970–88. doi: 10.1002/ece3.7674 (PMC8495813; doi:10.1002/ece3.7674)
Supplement: Supplementary file 1 — Supinfo S1 [file ECE3-11-12970-s001.docx]

APPENDIX


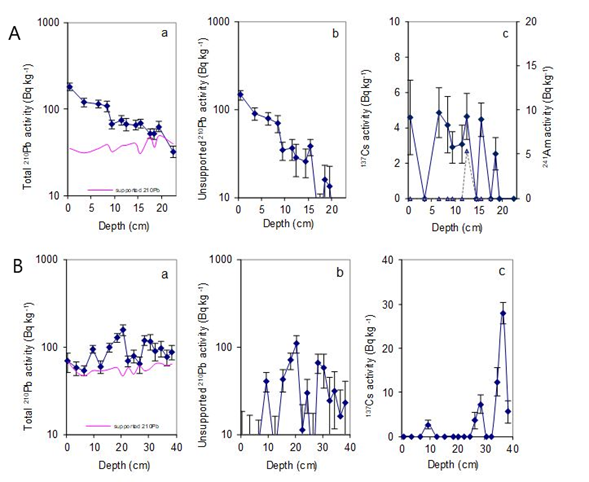


**FIGURE S1** Fallout radionuclide concentrations in (A) core LSAN1 (San Juana Lake); and (B) LBARB1 (Barbacoas Lake), showing (a) total ^210^Pb, (b) unsupported ^210^Pb, and (c) ^137^Cs and ^241^Am concentrations versus depth.

|  |
| --- |
|  |

**FIGURE S2** Temporal variation in geochemical elements found for LSAN1 (a) and LBARB1 (b) sediment cores.


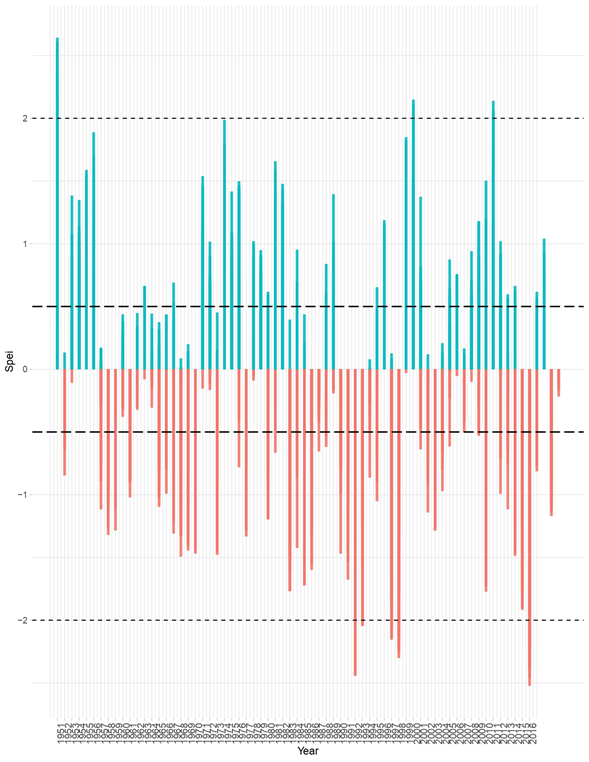


**FIGURE S3** SPEI analysis data for the identification of dry (red) and wet (blue) years during 1959-2016.

**FIGURE S4** Diatom stratigraphy from LSAN1 core. Major zones of compositional change are indicted by a red line and derived from *connis* clustering analysis.

**FIGURE S5** Diatom stratigraphy from LBARB1 core. Major zones of compositional change are indicted by a red line and derived from *connis* clustering analysis.

**FIGURE S5** MFA Dim 1 first derivatives (redline) of the fitted GAMs with confidence intervals (grey dotted line) for the San Juana Lake. The Blue line indicate significant increasing trends determined by the first derivative.

**TABLE S1** GAM statistical results for models used Gaussian distributions and identity link with the REML method. Basis dimension (k) results with low p-value (k-index<1) may indicate that k is too low, especially if edf is close to k'. A value of edf> 1 indicates non-lineal patterns whereas a value of 1 indicates a lineal relationship.

| **Lake** | **k'** | **edf** | **k-index** | **p-value** |
| --- | --- | --- | --- | --- |
| San Juana | 14.00 | 3.27 | 1.41 | 0.99 |
| Barbacoas | 14 | 1 | 1.07 | 0.49 |
